# Infection prevention and control without borders: comparison of guidelines on multidrug-resistant organisms in the northern Dutch-German cross-border region

**DOI:** 10.1186/s13756-025-01528-3

**Published:** 2025-02-12

**Authors:** Cansu Cimen, Matthijs S. Berends, Mariëtte Lokate, Corinna Glasner, Jörg Herrmann, Erik Bathoorn, Axel Hamprecht, Andreas Voss

**Affiliations:** 1 Institute of Medical Microbiology and Virology, University of Oldenburg, Oldenburg, Germany; 2 Department of Medical Microbiology and Infection Prevention, University Medical Center Groningen, University of Groningen, Groningen, The Netherlands; 3 Certe Medical Diagnostics and Advice Foundation, Department of Medical Epidemiology, Groningen, the Netherlands; 4 Institute for Hospital Hygiene Oldenburg, Klinikum Oldenburg, Oldenburg, Germany

**Keywords:** Vancomycin-resistant enterococci (VRE), Extended-spectrum β-lactamase *Enterobacterales* (ESBL-E), carbapenem-resistant *Enterobacterales* (CRE), Carbapenem-producing *enterobacterales* (CPE), Infection control, Guideline, Dutch-german cross-border region, The Netherlands, Germany

## Abstract

Infections due to multidrug-resistant organisms (MDROs) are a health threat due to increasing patient morbidity and mortality and the burden on healthcare systems. Robust infection prevention and control (IPC) measures are needed to minimize their emergence in hospitals. Therefore, various international and national IPC guidelines exist, yet the lack of harmonized IPC guidelines complicates the management of patients seeking healthcare across European borders. This study explores the similarities and differences in IPC measures for vancomycin-resistant enterococci (VRE) and multidrug-resistant (MDR) *Enterobacterales* both on local and national levels within the northern Dutch-German cross-border region. In Germany, IPC efforts are often led by hospital hygiene doctors, whereas in the Netherlands, they involve a collaboration between infection preventionists and clinical microbiologists, with local variations. The local guidelines in both countries, as expected, are based on national recommendations, yet introduce specific regulations in various aspects. The Dutch guidelines are more stringent for VRE management compared to the German guidelines, often imposing additional local measures beyond national requirements. The Dutch and German guidelines largely diverge in definitions of MDR Gram-negative bacteria. Unlike the Dutch guidelines, the German guidelines do not currently recommend screening or isolation for extended-spectrum beta-lactamase-producing *Enterobacterales*. For carbapenem-resistant and carbapenemase-producing *Enterobacterales*, there is no notable distinction between the countries’ guidelines, with both sharing the objective of maintaining a low prevalence and actively working towards containment. Inconsistencies in guidelines can lead to inefficient information exchange and inconsistent hygienic measures during patient transfers. Despite common commitments, differences in focus may reflect evolving understanding of MDRO transmission and ongoing debates on their management. Our findings highlight the divergence of IPC guidelines for the management of MDROs across two countries and call for collaboration in cross-border regions to increase the effectiveness of MDRO management in these regions and improve patient care.

**Supplementary Information:**

The online version contains supplementary material available at 10.1186/s13756-025-01528-3.

## Background

Multidrug-resistant organisms (MDROs) pose a significant threat to healthcare globally [1]. These organisms lead to severe healthcare-associated infections (HAI), prolonged hospital stays, increased healthcare costs, and higher mortality rates [2–4]. Moreover, patient referrals between hospitals and international travel play a significant role in MDROs dissemination and outbreaks across multiple institutions [5, 6]. In addition to robust infection prevention and control (IPC) measures, an interregional approach is found to be beneficial in optimizing preventive measures given the potential movement of patients seeking healthcare across international borders [7, 8].

Almost 40% of the European Union (EU) population lives in border regions. The Dutch-German cross-border, in particular, experiences the most frequent citizen exchanges [9]. Since 2005, the Dutch-German cross-border region has been collaborating to address antimicrobial resistance and IPC, facilitated by the European INTERREG programme [10]. These collaborative efforts could be relevant for the management of vancomycin-resistant enterococci (VRE) and multidrug-resistant *Enterobacterales* including extended-spectrum beta-lactamase-producing *Enterobacterales* (ESBL-E), carbapenem-resistant and carbapenemase-producing *Enterobacterales* (CRE and CPE, respectively), which have been ranked as high/critical-priority pathogens by the World Health Organization (WHO) [11]. In a study involving researchers and physicians from Germany and the Netherlands, the significance of gathering regional data for cross-border comparison was highlighted [12]. This approach was found crucial due to changing demographics in the border region, leading to increased demand for medical care, and therefore the increased risk of pathogen transmission among vulnerable individuals [12]. While disparities in older Dutch and German national IPC guidelines including local IPC guidelines of two university medical centres in the Netherlands and Germany have been documented previously [13], our focus extends to the local level in the northern Dutch-German cross-border region (Ems-Dollart region), where such distinctions remain underexplored, taking into account the new Dutch national guideline.

Our study aims to compare IPC measures for VRE and multidrug-resistant (MDR) *Enterobacterales* (ESBL-E and CRE/CPE) in hospitals on the local and national level, with a particular focus on screening methods, isolation measures, criteria for lifting isolation, readmission strategies, and recommended personal protective equipment (PPE) for healthcare workers (HCWs). By examining these aspects, we aim to establish a solid foundation for understanding and addressing the IPC challenges in the northern Dutch-German cross-border region.

## Methods

### Settings

The Ems-Dollart region is defined as the northern Dutch-German border region between the north-east of the Netherlands and the north-west of Germany, home to more than four million inhabitants. Two tertiary academic centers, the University Medical Center Groningen (UMCG) with 1339 beds, and the Klinikum Oldenburg (KOL), the largest hospital of the University Medicine Oldenburg with 830 beds, were selected for the local comparison within the region.

### Data extraction

We extracted information on screening, sampling sites, management of MDRO carriers, requirements for lifting isolation, and protocols for managing the readmission of an MDRO carrier and recommended PPE for HCWs regarding VRE and MDR *Enterobacterales*. This data was sourced from national guidelines provided by KRINKO (Kommission für Krankenhaushygiene und Infektionsprävention, Commission for Hospital Hygiene and Infection Prevention) [14, 15], the newly released MDRO-specific guideline of the SRI (Samenwerkingsverband Richtlijnen Infectiepreventie, Collaborative Infection Prevention Guidelines) [16], and local guidelines from the UMCG and KOL.

## Results

### Organizational structures of IPC practices

A comparison of the organizational structures of IPC practices between Dutch and German hospitals revealed marked differences. In Germany, “hospital hygiene” often exists as an independent department in university hospitals, with specifically trained infection control doctors (dedicated specialty) and infection control nurses leading IPC efforts. However, in the majority of the hospitals in Germany, infection control doctors serve as external consultants rather than employees and the infection control nurses work under the responsibility of the medical director of the hospital. In the Netherlands, by contrast, IPC is embedded within the medical microbiology department within most of the hospitals. IPC management involve collaboration between clinical microbiologists, who either have designated roles or a personal interest in the field, and infection preventionists, who typically have backgrounds in nursing, laboratory technology, or medical sciences and complete a dedicated IPC training.

In Germany, personnel and organizational requirements for IPC activities are mandatory nationwide. IPC staffing (IPC nurse, IPC doctor) recommendations in Germany are determined by the number of hospital beds and their categorization into three risk levels (A, B, C), with intensive care beds designated as the highest risk (category A) and regular wards as the lowest risk (category C) [17]. In the Netherlands, the current recommendations by professional societies are based on hospital admissions [18].

### Guidelines for the management of MDROs

Both the national and local IPC guidelines of the two university medical centres provide a comprehensive resource for managing MDROs in hospital settings. The local guidelines are primarily aligned with national recommendations, but also proactively introduce specific local regulations in various aspects.

When examining these four guidelines, the definition of VRE remains consistent across the border, though in the SRI-the Netherlands (SRI-NL) and UMCG guidelines, VRE consists only of *Enterococcus faecium*. However, in the case of multidrug-resistant (MDR) *Enterobacterales*, the Dutch and German guidelines diverge (Table S1, Table S2). The Dutch guidelines specify ESBL-E, CPE and CRE, while German guidelines use 3MRGN (multidrug-resistant Gram-negatives) and 4MRGN classification [12]. In detail, 3MRGN include strains resistant to three classes of antibiotics (piperacillin, cefotaxime/ceftazidime, and ciprofloxacin), encompassing ESBL-E strains with quinolone resistance, whereas 4MRGN signifies *Enterobacterales* resistant to four antibiotic classes and CPE/CRE. Detection and reporting of ESBL production in isolates without ciprofloxacin resistance is not mandatory based on the KRINKO-DE guideline, except in the neonatal intensive care units (ICUs) and paediatric wards [19]. In these wards, there is an additional category called “2MRGN NeoPaed” for Gram-negative bacteria that are resistant to ceftazidime and/or cefepime, since empirical treatment with fluoroquinolones cannot be administered to neonates and pediatric patients [19].

At the local level, the microbiology laboratory at KOL reports the presence of ESBL. Although this information is not used for IPC guidance, this approach is used for guiding antimicrobial decision-making when a patient develops infectious symptoms requiring antibiotic therapy.

### VRE-specific measures

SRI-NL and UMCG provides only measures for *E. faecium* based on VRE definition, while KRINKO-Germany (KRINKO-DE) and KOL guidelines are applicable for both *E. faecalis* and *E. faecium*.

KRINKO-DE provides a definition of ‘patients at risk for VRE’ and entrusts the decision to local IPC teams, focusing on the prevention of infections requiring antibiotic therapy. Similarly, KOL identifies specific risk groups and restricts screening to areas like haematology-oncology wards and ICUs (Table 1). However, both SRI-NL and UMCG guidelines have embraced a VRE screening algorithm that relies on individual patient characteristics without any risk ward categorisation (Table 1). Additionally, the UMCG has a regular screening program for all patients in the intensive care, haematology, and gastroenterology wards, regardless of their individual risk factors.

**Table 1:** Overview of national and local IPC measures for VRE in Germany and the Netherlands

*IPC measures*	*KRINKO-DE*	*KOL*	*SRI-NL* ^^^	*UMCG* ^^^
screening criteria	patients at risk for VRE^1^	• patients at risk are defined (bone-marrow transplant unit (BMT) and haematology-oncology ward) • contact patients that require isolation^3^	patients • with recent healthcare facility stays abroad • who had invasive procedures abroad • come from another Dutch healthcare facility with an ongoing VRE outbreak	• stricter than NL-SRI rules • patients at risk (ICU, haematology, gastroenterology) • additional recommendations for refugees/asylum seekers, adopted child, long-stay and dialyzed patients. • known / contact of VRE carrier
sampling site	rectal swab, stool	addition to KRINKO • urine in case of a urinary catheter • other previously positive sites (if applicable)	rectal swab, stool ^4^	same as SRI^6^
management of carriers	• contact isolation (single room)^1^ o for all OR o carriers at increased risk of environmental contamination^2^ • cohort^1^	addition to KRINKO • antiseptic whole-body washing	contact isolation (single room)	contact-plus isolation^7^
lifting the isolation	no recommendation	3 negative results on different days³ (1 week interval)	• 5 negative results^5^ (3 negatives suffice if PCR and cultivation are used) • follow-up cultures if admitted to an institution within 1 year after carrier status termination	3 negative results starting 1 year after the last positive culture
readmission measures of a known VRE patient	no recommendation	contact isolation for patients: • admitted to BMT or planning in next 6 weeks. • with VRE infection, diarrhoea, or faecal incontinence.	contact isolation if the patient found to be positive less than 1 year ago	addition to SRI recommends contact-plus isolation to patients with • last positive culture 1–5 years and > 5 years based on their number of negative cultures
recommended PPE for HCWs	gloves and gown	• only hand disinfection if no contact • long-sleeved gowns for direct contact • overcoat and trousers in case of very close contact.	gloves, long sleeve apron	same as SRI

*DE*,* Germany; HCW*,* healthcare worker; IPC*,* Infection Prevention and Control; KRINKO*, Kommission für Krankenhaushygiene und Infektionsprävention; *LVRE*,* linezolid-vancomycin resistant enterococci; NL*,* the Netherlands; PCR*,* polymerase chain reaction; PPE: personal protective equipment*,* SRI*,* Samenwerkingsverband Richtlijnen Infectiepreventie; UMCG*,* University Medical Center Groningen; KOL*,* Klinikum Oldenburg; VRE*,* vancomycin resistant enterococci*

^^^ valid only for *E. faecium*

^1^ decision should be taken by the clinicians, hospital hygienists and clinical microbiologists of the hospitals

^2^ insufficient compliances with hygienic measures, acute diarrhoea, faecal incontinence

^3^ patients on the BMT unit or oncology ward, VRE infection requiring treatment, VRE colonisation with presence of diarrhoea or faecal incontinence, evidence of LRE/LVRE (colonisation and/or infection)

^4^ additional sampling of other anatomical locations if needed: skin, throat, urine or wounds

^5^ cultures are not reliable when using antibiotics that suppress the growth of highly resistant microorganisms in the 48 h before collection

^6^ the following cultures are required in specific situations: sputum culture in intubated patients and in patients giving up sputum, smear of wounds and skin lesions (e.g. eczema or psoriasis), urine culture in patients with indwelling catheters or suspected urinary tract infection, umbilical smear in neonates (as long as the umbilical stump has not dried in)

^7^ cleaning and disinfection of the room and waste are handled differently, the patient lies in a sluiced room with the doors closed, allowing better differentiation between the clean and dirty zone

Screening for rectal carriage is the standard across all VRE guidelines. Additionally, SRI, KOL and UMCG acknowledge the possibility of taking samples from sites like, urine, or wounds, depending on the clinical situation (Table 1).

Furthermore, all four guidelines recommend contact isolation for VRE carriers, with UMCG introducing a contact-plus isolation terminology (an extra disinfection process is required for the patient room, and the doors are kept closed). While the Dutch guidelines do not leave much room for interpretation, the German guidelines recommend isolation only under circumstances. For instance, KRINKO-DE recommends that the IPC team evaluate the risk of environmental contamination to determine whether the patient should be isolated, whereas the KOL restricts isolation measures to specific high-risk wards.

Regarding lifting isolation for VRE carriers, KRINKO-DE offers no specific guidance while KOL suggests a minimum of three consecutive negative samples before considering terminating isolation. SRI-NL and UMCG require at least five consecutive negative results from one year after the last positive culture as a criterion.

The guidelines also diverge in their approach to re-admission measures of a known VRE carrier. KRINKO-DE does not provide a specific recommendation in this context, while SRI-NL recommends contact isolation if the VRE carrier’s positive test result is from the last year. The KOL bases its recommendation on patient risk groups, while the UMCG provides a detailed recommendation based on the number of negative results within a given time frame.

All guidelines recommend HCWs to wear gloves and gowns when approaching VRE-colonized/infected patients. The KOL recommends the use of overcoat and trousers, especially for situations involving very close contact, such as physiotherapy.

### MDR *Enterobacterales* specific measures

KRINKO-DE and KOL provide recommendations based on 3MRGN, 4MRGN, whereas SRI-NL and UMCG focus on recommendations on ESBL-E and CRE/CPE.

In general, the KOL guideline aligns with KRINKO-DE though it offers more detailed information in specific contexts regarding the screening and management of MDR *Enterobacterales.* KRINKO-DE does not recommend routine screening for 3MRGN but does for 4MRGN for patients with specific risk factors, while KOL recommends screening both for 3MRGN and 4MRGN in patients with higher risks for MDRO (Table 2).

**Table 2:** Overview of national and local IPC measures for MDR *enterobacterales* bacteria in Germany

*IPC measures*		*KRINKO-DE*	*KOL*
screening criteria		*3MRGN* not recommended *4MRGN* patients who had • recent healthcare contact in 4MRGN-endemic countries. • contact with 4MRGN-positive patients. • inpatient stay (> 3 days) in a high 4MRGN prevalence region within the past 12 months.	• patients o with positive history o transferred from foreign hospitals. • immigrants from emergency reception centres
sampling site		rectal (wound and urine if needed)	rectal, urinary tract catheter, urine and known localization of the MRGN
management of carriers		*3MRGN* contact precaution (single room/ cohort): only valid for *E. coli* and *K. pneumoniae* at risk wards^1^ *4MRGN* contact isolation at all hospital wards	*3MRGN* same as KRINKO^2,3^ *4MRGN* contact-plus isolation
lifting the isolation		no recommendation	• 3 negative results on different days (1 week interval) • weekly control series after lifting the isolation
readmission of a known MDRGN patient to a normal ward			*3MRGN* uptake screening and basic hygiene *4MRGN* uptake screening and isolation
readmission of a known MDRGN patient to risk-wards			uptake screening and isolation
recommended PPE for HCWs		gloves and long-sleeved gown	• same as KRINKO for direct contact • only hand disinfection if no contact. • overcoat and trousers for very close contact. • triple-layer medical mask if evidence of respiratory tract colonisation and direct patient contact.

*DE*,* Germany; HCW*,* healthcare worker; KRINKO*, Kommission für Krankenhaushygiene und Infektionsprävention; *MRGN*,* multidrug-resistant Gram-negative bacteria; KOL*,* Klinikum Oldenburg*

^1^ Risk areas are defined after individual risk assessment depending based on the patient’s material and structural conditions. Intensive care wards, neonatology, and haematological-oncological wards are risk wards. For *Serratia* spp., isolation is recommended in neonatology wards

^2^ intensive care unit, dialysis, oncology and BMT

^3^ cohort isolation can only be carried out for patients with an MRGN of the same pattern (if necessary, consultation with the hygiene team)

Contact isolation is advised for 4MRGN in all hospital wards, whereas for 3MRGN, it is specifically applicable to *E. coli* and *K. pneumoniae* only in at-risk wards (Table 2). KRINKO-DE does not have further recommendation for the management of MDR *Enterobacterales*. KOL recommends at least three negative samples taken to lift the isolation both for 3MRGN and 4MRGN and weekly control series are recommended till the end of hospital stay. While the recommendations change depending on whether a patient who is previously known to be a carrier of 3MRGN is taken to the normal or high-risk ward, the same is recommended for 4MRGN in all departments of the hospital. Both guidelines recommend gloves and long-sleeved gown while KOL elaborate the recommendations for PPE for HCPs according to different scenarios.

Generally, the UMCG guideline offers more intricate and stringent recommendations for the screening and management of MDR *Enterobacterales* compared to the SRI-NL. Regarding the screening criteria for ESBL-E and CRE/CPE, SRI-NL focuses on the patients coming from abroad, while UMCG has additional rules for the patients coming from another Dutch hospital (Table 3). Both guidelines recommend rectal screening. Both guidelines advise contact isolation for ESBL-E; SRI gives flexibility for ESBL-E patients to keep in multiple patient rooms with some conditions, whereas UMCG recommends island nursing for ESBL producing *E. coli*. For CPE/CRE, SRI-NL recommends contact isolation whereas UMCGUMCG recommends contact-plus isolation (the patient lies in a sluiced room with the doors closed, allowing better differentiation between the clean and dirty zone). SRI-NL recommends lifting isolation after two negative cultures: starting three months after the last positive culture for ESBL-E and starting one year after the last positive culture for CRE/CPE. UMCG follows these guidelines but recommends the same one-year period for ESBL *K. pneumoniae* as for CRE/CPE. There is no specific recommendation for the readmission of a known MDR *Enterobacterales* carrier in the SRI-NL guideline, though UMCG describes the screening/isolation rules regarding the timing of the last recorded positive culture. Both SRI-NL and UMCG recommend gloves and a long-sleeved apron for HCWs.

**Table 3:** Overview of national and local IPC measures for multidrug-resistant Gram-negative bacteria in the Netherlands

IPC measures	*SRI-NL*	*UMCG*
screening criteria	patients • ^1^with recent healthcare facility stays abroad • who had invasive procedures abroad • come from another Dutch healthcare facility with an ongoing uncontrolled outbreak • lived in a refugee shelter < 2 months ago.	• stricter with Dutch hospital rules (screening regardless of an ongoing outbreak) • additional recommendations for refugees/asylum seekers, adopted child, long-stay and dialyzed patients. • known / contact of ESBL-E/CPE/CRE carrier.
sampling site	rectum/perirectum/faeces sample	^2^rectum
management of carriers	*ESBL-E* • contact isolation • contact precautions in multiple rooms allowed if no single room is available, with a 1.5 m bed clearance *CRE/CPE* contact isolation	*ESBL-E* • contact isolation • ESBL-producing *E. coli*: island nursing *CRE/CPE* ^3^contact-plus isolation
lifting the isolation	*ESBL-E* 2 negative results starting 3 months after the last positive culture *CRE/CPE* • 2 negatives starting 1 year after the last positive culture • Follow-up cultures for admissions in the first year after carrier status termination	*ESBL-E* ^4^same as SRI *CRE/CPE* same as SRI
readmission of a known MDRGN patient	No recommendation	*ESBL-E* procedures differ between ESBL-producing *E. coli* and the other *Enterobacterales* for declaring negative, lifting isolation based on the number of known negatives and the time since the last positive culture *CRE/CPE* • uptake screening and isolation • no difference among different microorganisms • conditions for lifting the isolation are stricter
recommended PPE for HCWs	gloves, long-sleeved apron	same as SRI

*HCW*,* healthcare worker; NL*,* the Netherlands; PCR*,* polymerase chain reaction; PPE*,* personal protective equipment; SRI*,* Samenwerkingsverband Richtlijnen Infectiepreventie; UMCG*,* University Medical Center Groningen*

^1^ for the individuals who have been to Asia and/or Africa < 2 months ago without healthcare facility stays: healthcare institutions are recommended to choose to include this group in the risk inventory depending on the local situation

^2^ in case of specific situation: sputum culture in intubated patients and in patients giving up sputum, smear of wounds and skin lesions (e.g., eczema or psoriasis), urine culture in patients with indwelling catheters or suspected urinary tract infection, umbilical smear in neonates (as long as the umbilical stump has not dried in)

^3^ cleaning and disinfection of the room and waste are handled differently, the patient lies in a sluiced room with the doors closed, allowing better differentiation between the clean and dirty zone

^4^for ESBL *K. pneumoniae*: 2 negatives starting 1 year after the last positive culture

## Discussion

In this comparative study, we reveal significant differences not only between the national IPC guidelines for managing MDROs in the Netherlands and Germany, but also when comparing national and local guidelines from two hospitals from the northern Dutch-German cross-border region. The differences may vary depending on the local epidemiology of the MDROs involved and the diagnostic methods used, as well as on the resources and healthcare structures of individual hospitals. Although Germany and the Netherlands share a common socio-cultural background and both have well-established healthcare systems, there are important differences in political centralization, history, healthcare structures and staffing [20], that may influence the organization of IPC guidelines [8]. In this context, the Cross-Border Institute (CBI), an initiative of the University of Groningen / Aletta Jacobs School of Public Health, the University Medical Center Groningen and the University of Oldenburg, draws attention to the importance of cooperation in health within the Ems-Dollart region including IPC by highlighting the different healthcare structures between these two countries and learning from each other best practices [21].

We observed that the distinction between national and local guidelines is more pronounced in Germany than in the Netherlands, given that KRINKO-DE only makes general recommendations for both VRE and MDRGN or no recommendations at all, such as lifting isolation or readmission of a patient known to be an MDRO carrier. This observation can be attributed to the differences in political governance and degree of centralization between these two countries. In the Netherlands, where political centralization is more prominent, national guidance has a significant influence. In contrast, for Germany, a federally organized country, the federal states have the autonomy to adapt these guidelines locally.

### VRE-specific measures

The definition of VRE remains consistent in both countries, suggesting potential collaboration both at local and national level. Yet, the Dutch guidelines demonstrate a higher level of stringency compared to the German guidelines for VRE management, with local guidelines imposing additional measures beyond the national requirements.

There are two notable distinctions between the Dutch and German guidelines regarding VRE. Firstly, the German guidelines focus VRE screening on patients in high-risk wards, whereas the Dutch guidelines do not differentiate between high-risk and normal care wards. Instead, VRE screening in the Dutch guidelines is based on patient-specific risk factors. Secondly, the German guidelines prioritise preventing infections that require antibiotic therapy by categorising patient groups based on their risk of developing VRE infection and apply hygiene measures accordingly. In contrast, the Dutch guidelines advocate for a strategy focused on searching, detecting, and isolating cases.

The substantial differences may be attributed to the mixed evidence on the effectiveness of VRE screening and isolation of the carriers [22–25]. Yet, the prevalence of MDROs in hospitals may serve as a metric for assessing the scope and efficacy of IPC measures. The epidemiological situation of elevated VRE prevalence in German hospitals presents a stark contrast to the consistently low prevalence observed in the Dutch hospitals over the last ten years [26]. In addition to nationwide prevalence differences, a large prevalence study conducted in 23 hospitals within the German-Dutch border region found that the prevalence of rectal VRE colonisation in intensive care unit patients was nearly 30 times higher in German hospitals (2.7%) compared to Dutch hospitals (0.1%) [26, 27]. Since transmission of VRE primarily leads to patient colonisation, with a low risk of developing infection in these patients [28], the absence of systematic screening policies allows transmission to go undetected and spread of VRE within and across healthcare facilities [29]. This might be one potential explanation for the high prevalence of VRE in German hospitals, emphasizing the importance of robust IPC strategies to reduce the spread of VRE.

From another perspective, the differences in screening and isolation guidelines for VRE can also be explained by the variation in epidemiology between the two countries. Both national IPC guidelines mention the importance of considering the country’s epidemiology of the respective MDRO when determining which measures to apply [14, 16]. For example, KRINKO-DE states that an endemic situation for VRE exists in many regions of Germany, so that a higher proportion of patients are already colonised when they are admitted to a hospital and it would thus be hardly possible to prevent transmission of VRE with implemented measures [14]. This reasoning is based on expert opinion and supported by the studies [30, 31]. In contrast, the SRI-NL grounds the decision of comprehensive VRE screening and isolation of VRE cases on the low epidemiology of VRE in the Netherlands [27, 32]. One could argue that decisions are made based on the existing epidemiology; however, this raises a fundamental causality dilemma: whether epidemiology drives the guidelines, or the guidelines influence the epidemiology.

Furthermore, the German national KRINKO-DE guidelines are typically adaptable to the specific epidemiological conditions within hospitals and regions, rather than providing rigid, fixed rules for screening, which is quite the opposite of the Dutch national SRI-NL guidelines. The flexibility of the KRINKO-DE guidelines may be attributed to the varying epidemiological landscape of VRE across Germany [33]. Assessing this situation across Germany, it is evident from other studies that Lower Saxony exhibits a lower prevalence of VRE compared to the rest of the country [33, 34]. Yet, despite VRE prevalence in Lower Saxony being lower than the national average in Germany, a Dutch-German cross-border comparison indicates a higher incidence in Lower Saxony compared to the northern region of the Netherlands [35]. In this study conducted in Ems-Dollart region, the prevalence of patients colonized with VRE was found to be higher in the KOL than the UMCG the Ems-Dollart region [35]. This difference becomes more noteworthy when considering that the screening criteria, guidelines for lifting isolation, and the management of readmission for known VRE patients are more lenient at the KOL than the UMCG.

Regarding the decision to lift the isolation for VRE colonised patients, the two national guidelines base their recommendations on different publications. KRINKO-DE does not have specific recommendations on lifting isolation and argues the fact that the colonisation period for VRE has been reported to range from weeks to more than three years, making it impossible to establish a definitive duration [36–38]. In addition, KRINKO-DE highlights the limitation of detecting VRE with rectal swabs by citing a study that found in patients with a hospital stay more than 30 days, VRE was no longer detectable in 18% of VRE carriers after an average stay of 26 days [39]. This finding strengthens the argument against recommending a definitive duration for lifting isolation. Differing from the German approach, SRI-NL also acknowledges the ‘long-term carrier’ situation for VRE and the potential false-negative culture results but emphasises the significant risk of VRE spread in healthcare institutions. Therefore, instead of avoiding recommendation, the SRI-NL takes the initiative to consider a carrier as positive during the first year after the last positive culture, even if there are negative cultures in between. Furthermore, the recommendation to lift isolation after five consecutive negative cultures is based on a study that shows its high reliability in confirming that carriage has ended [40]. SRI-NL extends the recommendation further, based on a study that has shown the increased reliability of molecular diagnostics over conventional swab cultures in confirming the end of VRE carriage [41]. These observations suggest that the guidelines are based on the different body of evidence.

### MDR *Enterobacterales* - specific measures

Regarding MDR *Enterobacterales*-specific differences, it is important to firstly note that the definitions of these pathogens differ widely between the Netherlands and Germany [12, 42]. The main distinction lies in the assessment of ESBL-E. In Germany, ESBL-E are neither separately classified nor is their detection mandated in the national guideline, except in the neonatal ICUs and paediatric wards [19]. In a study comparing antibiograms of Gram-negative bacteria based on the national classification systems, it was demonstrated that less ESBL-E isolates were flagged according to the 3MRGN classification as expected, leading to them not being classified as MDRO [42, 43].

As ESBL-E is not separately classified, the German guidelines do not currently recommend screening for ESBL-E. This approach may be perceived as a drawback compared to the Dutch guidelines, which recommends patient specific risk-based screening for ESBL-E. While universal screening for ESBL-E remains controversial due to uncertainty about its effectiveness in reducing nosocomial transmission, particularly in endemic settings [44, 45], screening efforts towards individuals at a high risk of ESBL-E colonisation could be considered due to the increased risk for developing infections in patients colonized with ESBL-E [28, 46–48].

Both KRINKO-DE and SRI-NL guidelines base their expert recommendations on the literature, yet they rely on different studies. It has to be taken into account that KRINKO guideline was published in 2012, whereas SRI-NL was published in 2024 and could therefore accommodate the better body of evidence today. KRINKO-DE bases its recommendation against screening for third-generation-cephalosporin-resistant *Enterobacterales* (3GCREB) or ESBL-E on three main publications published between 2002 and 2007. A cohort study conducted among organ transplant recipients revealed a high 3GCREB colonisation rate (24%) but minimal patient-to-patient transmission, concluding that screening and isolating such cases in an endemic setting is inefficient [49]. Another study conducted in ICUs found a low prevalence of ESBL-E carriers upon admission (0.97%) and detected 15 out of 28 cases simultaneously through clinical samples, concluding the routine screening program was not cost-effective in a non-epidemic setting [50]. The third study on surveillance reports reported an incidence of 0.12 ESBL-E cases per 1000 patient days, indicating an endemic setting [51]. The authors concluded that contact isolation alone may not prevent spread in such settings, but emphasized the importance of ESBL-E as a cause of hospital-acquired infections and recommended continued barrier precautions for high-risk populations [51]. The German approach of not screening or isolating ESBL-E cases may also be explained by the high prevalence (up to 12.7%) of 3GCREB and ESBL-E reported in several studies conducted in German hospitals [52–56]. One study performed admission screening in 4376 patients from six centres and assessed risk factors for 3GREB carriage [53]. A prevalence of 9.5% 3GCREB was reported and five risk factors for carriage were identified [53]. However, 79.3% of all colonized patients were positive for at least one risk factors, but also 61.7% of all patients not colonized with 3GCREB [53]. This casts doubts on the efficiency of a risk factor based screening program, especially in settings with a high prevalence of 3GCREB [53]. Furthermore, in hospital transmission of ESBL-E has been shown to be a very rare event [57]. In contrast, the SRI-NL recommends vertical prevention measures despite of the considerable background prevalence of ESBL-E carriage in the Netherlands, which is 6.2% in a current study [58]. SRI-NL advocates targeted screening on admission for patients with risk factors associated with increased prevalence of ESBL-E carriage compared to the Dutch population, such as patients treated in foreign hospitals [59, 60] or patients who have been in a refugee shelter [61, 62].

The absence of ESBL-E screening in German guidelines leads to a lack of precautions except for 3MRGN in risk wards, while the Dutch guidelines provide preventive measures for all ESBL-E followed by screening. Both the SRI-NL and UMCG guidelines recommend contact precautions for all ESBL-E cases, consistent with evidence from a randomized clinical trial permitting isolation or cohorting [63]. In addition to the epidemiological situation discussed above, another explanation to the German approach of not screening or isolating ESBL-E cases could be the recognised limited capacity of ESBL-E, particularly *E. coli*, to spread within healthcare environments [46, 57, 64, 65]. However, it’s notable that the Dutch guidelines have not disregarded these factors but have instead adapted their preventive measures accordingly. For instance, the UMCG guideline permits island nursing for ESBL-producing *E. coli*, while the SRI-NL guideline allows cohorting in case no single room is available. This adaptation reflects the diverse approaches seen recently in Dutch hospitals, where differences in molecular epidemiology and spread between ESBL-E prompt tailored strategies [12].

Concerning the definition of CRE/CPE, there is no notable distinction between countries or the hospitals, given that CRE/CPE is used in the Dutch guidelines and 4MRGN in the German guidelines, including all isolates with resistance to carbapenems, but also all carbapenemase producers. Outcomes from a recent study, using various definitions, indicate that the ‘KRINKO-4MRGN’ criteria effectively identify all CRE cases [66], suggesting there are no downsides to using these criteria for preventive measures for *Enterobacterales*.

Furthermore, although there are differences for the patients recommended to be screened, all four guidelines commonly recommend contact isolation in a single room for CRE/CPE. This corresponds with the shared objective in both countries to maintain a low prevalence of CRE/CPE and actively work towards its containment [67]. Although the KRINKO-DE guidelines do not include specific details about lifting isolation, and none of the national guidelines provide recommendations on managing known CRE patients, both local guidelines tend to address these conditions despite varying rules. This consistency at the local level creates a unique environment that differs from national standards but allows for easier cooperation within the region.

### Challenges and prospects

Patient transfer between countries is a risk factor for the spread of MDROs [68, 69]. The discrepancies in guidelines increase the likelihood of inefficient information exchange and inconsistent hygienic precautions during these transfers and the need for close cooperation becomes more pronounced in cross-border regions. We encountered difficulties even in the comparative analysis of IPC guidelines, especially due to the difference in definition of MDR *Enterobacterales*. Thus, these differences pose a challenge for a unified and effective response in the Ems-Dollart region, or potentially to that end, any cross-border region.

On the other hand, adapting protocols will not only enable smoother patient management and effective information exchange but also lay the groundwork for potential standardized cross-border notifications and promote improved cooperation in combating MDROs across the region. For example, the previous INTERREG MRSA-Net and EurHealth-1Health projects demonstrated that adapted screening strategies and standardized care within cross-border networks could reduce regional MRSA prevalence [70, 71].

### Strengths and limitations of the study

Comparing the local guidelines of two tertiary care hospitals in a cross-border region alongside the national guidelines of the respective countries provided a comprehensive understanding of IPC healthcare practices in that region. It allowed for the identification of differences not only between the national guidelines of the two countries but also between the national and local guidelines within each country.

Our study intentionally centres on a comparative analysis of two tertiary care medical centres. While this approach offers valuable insights specific to tertiary care settings, it naturally does not encompass the broader spectrum of secondary care hospitals and exploring these additional healthcare facilities could enrich our understanding of challenges.

## Conclusion

Our comparative analysis of IPC guidelines for MDROs, with the focus on VRE and ESBL-E and CPE/CRE, between the Netherlands and Germany, especially in the context of cross-border regions, highlights important differences in both national and local approaches. These differences may arise from a variety of factors, including political governance, healthcare structures, local epidemiology and time of publication/available evidence. Despite many commonalities, differences in focus may reflect the evolving understanding of the transmission of the mentioned MDROs and the ongoing debate surrounding their management. While the challenges in harmonizing cross-border guidelines are clear, our findings call for collaboration in cross-border regions. Therefore, understanding the differences, learning from each other’s strengths by adapting IPC efforts to local circumstances are of utmost importance to effectively combat the spread of MDROs and secure patient care internationally, across existing healthcare structures.

## Electronic supplementary material

Below is the link to the electronic supplementary material.

Supplementary Material 1

## Data Availability

No datasets were generated or analysed during the current study.

## Abbreviations

CBI: Cross-Border Institute
CPE: Carbapenemase-producing Enterobacterales
CRE: Carbapenem-resistant Enterobacterales
ESBL-E: Extended-spectrum beta-lactamase-producing Enterobacterales
EU: European Union
HAI: Healthcare-associated infections
HCWs: Healthcare workers
IPC: Infection prevention and control
KRINKO: Kommission für Krankenhaushygiene und Infektionsprävention, Commission for Hospital Hygiene and Infection Prevention
KOL: Klinikum Oldenburg
MDROs: Multidrug-resistant organisms
MRGN: Multidrug-resistant Gram-negatives
PPE: Personal protective equipment
SRI: Samenwerkingsverband Richtlijnen Infectiepreventie, Collaborative Infection Prevention Guidelines
UMCG: University Medical Center Groningen
VRE: Vancomycin-resistant enterococci
