# Supplementary material for: Infection prevention and control without borders: comparison of guidelines on multidrug-resistant organisms in the northern Dutch-German cross-border region
Source: Antimicrob Resist Infect Control. 2025 Feb 12;14:11. doi: 10.1186/s13756-025-01528-3 (PMC11817605; doi:10.1186/s13756-025-01528-3)
Supplement: Supplementary file 1 — Supplementary Material 1 [file 13756_2025_1528_MOESM1_ESM.docx]

**Table S1.** Classification of multidrug-resistant *Enterobacterales* according to German guideline

| **Antibiotic/resistance mechanism** |  | **3MRGN** | **4MRGN** |  |
| --- | --- | --- | --- | --- |
| Piperacillin |  | R | R |  |
| Cefotaxime/ceftazidime |  | R | R |  |
| Ciprofloxacin |  | R | R |  |
| Imipenem/meropenem |  | S | R |  |
| Carbapenemase positive |  |  | ✓ |  |

MRGN, multidrug-resistant Gram-negatives.

MRGN is based on the phenotypic susceptibility testing to four antibiotic classes. 3MRGN means resistance to 3 antibiotic classes and 4MRGN means resistance to 4 antibiotic classes. In case of carbapenemase detection in the isolate irrespectively of the resistance phenotype, it is classified as 4MRGN.

**Table S2.** Classification of multidrug-resistant *Enterobacterales* according to Dutch guideline

| **Antibiotic/resistance mechanism** |  | **BRMO** |
| --- | --- | --- |
| Fluoroquinolones and aminoglycosides and  co-trimoxazole (MDR-*Enterobacterales* group II) |  | R |
| ESBL positive |  | ✓ |
| Carbapenemase positive or carbapenem resistant |  | ✓ |

BRMO, bijzonder resistente micro-organismen (particular resistant micro-organisms); ESBL, Extended-spectrum beta-lactamases; MDR, multidrug-resistant.

Resistance to fluoroquinolones and aminoglycosides and co-trimoxazole OR being ESBL positive OR being carbapenemase positive or resistance to carbapenems is considered to be BRMO.

**Table S3.** Overview of national and local IPC measures for VRE

| IPC measures | KRINKO-DE | KOL | SRI-NL^^^ | UMCG^^^ |
| --- | --- | --- | --- | --- |
| screening criteria | - patients at risk for VRE^1^ (three samples on different days over a period of at least one week (days 2, 5 and 7) | - bone-marrow transplant unit (BMT) and haematology-oncology ward (at admission and weekly) - contact patients that require isolation^2^ (3 smear series (day 0, 7, 14) | - patients who spent more than 24 hours in a healthcare facility abroad less than two months ago. - patients who stayed less than 24 hours in a foreign healthcare institution less than two months ago and who have had an invasive procedure in the foreign healthcare institution. - patients who stayed in a healthcare facility abroad more than two months ago and who have had an invasive procedure in the foreign healthcare facility. - patients who come from another Dutch healthcare institution from a department where there is a VRE outbreak, and which is not yet under control. | - immediate readmission after a stay of more than 24 hours in a Dutch hospital - patients taken over from another Dutch/foreign hospital. - admission to a foreign hospital within the last year^4^ - refugees/asylum seekers^5^ (arrived within the last three years) - adopted child within the last year. - long-stay patients (every 2 weeks) - guest dialyzer from abroad in case of long dialysis period. - dialyze patient who temporarily dialyzed abroad. - known / contact of VRE carrier |
| sampling site | - rectal swab, stool | - rectal swab (visible faecal material required), stool. - urine in case of a urinary catheter - other previously positive sites (if applicable) | - rectal swab, stool | - rectal/faecal swab^6^ |
| management of carriers | - contact isolation (single room)^1^   - for all OR   - carriers at increased risk of environmental contamination (insufficient compliance with hygienic measures, acute diarrhoea, faecal incontinence) - cohort^1^ | - contact isolation (single room)^2^ - only genetically identical VRE cases can be cohorted (after consultation with hospital hygiene team) - antiseptic whole-body washing | - contact isolation (single room) | - contact-plus isolation^7^ |
| lifting the isolation | no recommendation | - at least 3 negative samples taken on different days (at least 1 week interval) - negative swab results under antibiotic therapy can only be used to a limited extent.   - control after the end of the antibiotic therapy by smear series | - determine the end of VRE carriage based on   - 5 negative samples^3^ from rectum/perirectum/faeces, and if applicable also from other sampling sites where VRE has been previously found.   - collected on different days from 1 year after first positive culture. - If PCR and cultivation are used in the detection, 3 negative samples are sufficient. - Take follow-up cultures if a person is admitted to an institution in the first year after termination of carrier status to check for recurrences. | - not lifted until discharge of the patient |
| readmission measures of a known VRE patient | no recommendation | - if the patient is not admitted to BMT or not planning in the next 6 weeks: basic hygiene. - if the patient is admitted to BMT or planning in the next 6 weeks AND   - there is a negative smear series that is not older than 6 weeks at the time of admission: basic hygiene.   - if not:     - isolate or cohort (with hospital hygiene consultation)     - take VRE swab series:       - negative: lift the isolation and basic hygiene. - screen the patient if the patient has:   - VRE infection   - diarrhoea   - faecal incontinence     - negative: basic hygiene     - positive: isolation       - lift the isolation if the patient is VRE free for 1 year (3 negative series in distance of at least 4 weeks) with hospital hygiene consultation. | - contact isolation if the patient found to be positive less than 1 year. | - last positive culture <1 year   - contact-plus isolation and admission culture   - weekly cultures until there are 5 negative cultures.   - do not lift the isolation within 1 year. - last positive culture 1-5 years, <5 negative cultures   - contact-plus isolation and admission culture   - weekly cultures until there are 5 negative cultures.   - lift the isolation at 5 negative cultures. - last positive culture 1-5 years ago and ≥5 negative cultures   - admission culture   - after negative uptake culture declare negative - last positive culture ≥5 years ago and <5 negative cultures   - admission culture   - 2 negative cultures (including 1 in current admission): declare negative. - last positive culture ≥5 years ago and ≥5 negative cultures   - declare it negative. |
| recommended PPE for HCWs | gloves and gown | - long-sleeved gowns for direct patient contact - overcoat and trousers in case of very close contact - only hand disinfection in case no contact at all | - gloves, long sleeve apron | gloves and disposable gown |

*DE, Germany; HCW, healthcare worker; IPC, Infection Prevention and Control; KRINKO,* Kommission für Krankenhaushygiene und Infektionsprävention; *LVRE, linezolid-vancomycin resistant enterococci; NL, the Netherlands; NA, not applicable; PCR, polymerase chain reaction; PPE: personal protective equipment, SRI, Samenwerkingsverband Richtlijnen Infectiepreventie; UMCG, University Medical Center Groningen; UOL, University Oldenburg Hospital; VRE, vancomycin resistant enterococci.*

^^^ valid only for *E. faecium*

^1^ decision should be taken by the clinicians, hospital hygienists and clinical microbiologists of the hospitals.

^2^ patients on the BMT unit or oncology ward, VRE infection requiring treatment, VRE colonisation with presence of diarrhoea or faecal incontinence, evidence of LRE/LVRE (colonization and/or infection)

^3^ cultures are not reliable when using antibiotics that suppress the growth of BRMO in the 48 hours before collection.

^4^ only apply if the patient was admitted for more than 24 h or less than 24h if an invasive procedure or risk factor was present.

^5^ as long as patients live an asylum centre, cultures are repeated every 2 months.

^6^ in case of specific situation: Sputum culture in intubated patients and in patients giving up sputum, Smear of wounds and skin lesions (e.g., eczema or psoriasis), Urine culture in patients with indwelling catheters or suspected urinary tract infection, Umbilical smear in neonates (if the umbilical stump has not dried in)

^7^ cleaning and disinfection of the room and waste are handled differently. the patient lies in a sluiced room with the doors closed, allowing better differentiation between the clean and dirty zone.

Conditions for lifting isolation in case readmission at UMCG: Only cultures taken when the patient is not on antibiotics are reliable. If the patient is on antibiotics, valid cultures can be taken at least 48 hours after stopping antibiotics. Subsequent BRMO cultures should be taken at least 24 hours apart. If a patient's readmission cultures are positive, protective measures during the relevant admission are not lifted.

**Table S4.** Overview of national and local IPC measures for multidrug-resistant *Enterobacterales*

|  | KRINKO-DE | | KOL | |
| --- | --- | --- | --- | --- |
| IPC measures | 3MRGN | 4MRGN | 3MRGN | 4MRGN |
| screening criteria | - not recommended | - patients with recent contact with the health care system in 4MRGN endemic countries - patients who have had contact with 4MRGN positive patients.   Patients with an inpatient hospital stay (> 3days) in the past 12 months in a region with an increased 4MRGN prevalence | - positive history - patients transferred from foreign hospitals. - immigrants from emergency reception centres | |
| sampling site | - rectal (wound and urine if needed) | | - rectal | |
| management of carriers | - contact isolation (single room or cohort)   only valid for *E. coli* and *K. pneumoniae* at risk areas^1^ | - contact isolation (single room or cohort)   - at all hospital areas | - contact isolation (single room)   - only valid for *E. coli* and *K. pneumoniae* at risk areas^2^ - cohort isolation can only be carried out for patients with an MRGN of the same pattern (if necessary, consultation with the hygiene team) - decolonization is not recommended (can be evaluated at the case level) | - contact isolation (single room)   - at all hospital areas - cohort isolation can only be carried out for patients with an MRGN of the same pattern (if necessary, consultation with the hygiene team) - decolonization is not recommended (can be evaluated at the case level) |
| lifting the isolation | NA | | - at least 3 negative samples taken on different days (at least 1 week interval) - after the isolation is removed, a control series is applied once a week for the rest of the hospital stay. - Negative swab results under antibiotic therapy can only be used to a limited extent (control after the end of the antibiotic therapy using by means of a series of swabs). | |
| readmission of a known MDRGN patient to a normal ward | NA | | - 1 smear series (rectal, urinary tract catheter, urine and known localization of the 3MRGN)   positive or negative: basic hygiene | - uptake screening § isolation - 3 smear series on three consecutive days (rectal, urinary tract catheter, urine and known localization of the 4MRGN)   - negative: lift the isolation and check weekly.   positive: continue isolation. |
| readmission of a known MDRGN patient to at risk-wards | NA | | - uptake screening and isolation - 3 smear series on three consecutive days (rectal, urinary tract catheter, urine and known localization of the MRGN)   - negative: lift the isolation and check weekly. - positive: continue isolation | |
| recommended PPE for HCWs | gloves and long-sleeved gown | | - gloves - long-sleeved gowns for direct patient contact - overcoat and trousers in case of very close contact - only hand disinfection in case no contact at all - triple layer medical mask: wear mouth and nose protection only if there is evidence of colonization in the respiratory tract and in direct patient contact | |

**Table S5.** Overview of national and local IPC measures for multidrug-resistant Gram-negative bacteria

|  | SRI-NL | | UMCG | |
| --- | --- | --- | --- | --- |
| IPC measures | ESBL-E | CPE/CRE | ESBL-E | CPE/CRE |
| screening criteria | - ^1^patients who spent more than 24 hours in a healthcare facility abroad less than two months ago. - patients who stayed less than 24 hours in a foreign healthcare institution less than two months ago and who have had an invasive procedure in the foreign healthcare institution. - patients who stayed in a healthcare facility abroad more than two months ago and who have had an invasive procedure in the foreign healthcare facility. - patients who come from another Dutch healthcare institution from a department where there is an outbreak, and which is not yet under control. - patients who have lived in a refugee shelter less than two months ago. - patients who have been to Asia (including Turkey) and/or Africa less than two months ago. | | - immediate readmission after a stay of more than 24 hours in a Dutch hospital - patients taken over from another Dutch/foreign hospital. - admission to a foreign hospital within the last year^4^ - refugees/asylum seekers^5^ (arrived within the last three years) - adopted child within the last year. - long-stay patients (every 2 weeks) - guest dialyzer from abroad in case of long dialysis period. - dialyze patient who temporarily dialyzed abroad. - known / contact of ESBL-E/CPE/CRE carrier. | |
| sampling site | - rectum/perirectum/ feces sample | | - rectum^6^ | |
| management of carriers | - contact isolation (single room)^2^ | | - contact isolation. - ESBL producing *E. coli*: island nursing. | - contact-plus isolation^7^ |
| lifting the isolation | - determine the end of carriage based on   - 2 negative (peri-)rectal or fecal cultures^3^, and if applicable also from other sampling sites where the ESBL-E was previously found.   - collected on different days from 3 months after the last positive culture | - Determine the end of carriage based on   - 2 negative samples^3^ from rectum/perirectum/ faeces, and if applicable also from other sampling sites where the CRE has been previously found.   - collected on different days from 1 year after the last positive culture. - Take follow-up cultures if a person is admitted to an institution in the first year after termination of carrier status to check for recurrences. | - after ≥5 negative cultures (not taken under antibiotics AND at least 24h apart). | - not lifted until discharge of the patient. |
| readmission of a known MDRGN patient | NA | | ESBL producing *E. coli:*   - Last positive culture ≤2 years ago and no negative cultures known.   - island nursing + admission culture 2nd culture after 24h   - declare negative if case of 2 negative cultures. - Last positive culture ≤2 years ago and 1 negative culture known.   - island nursing + admission culture   - declare negative in case of negative admission culture. - Last positive >2 years ago   - declare negative. - ≥2 negative cultures of which at least 1 from now   The rest:   - Last positive culture < 1 year ago and there are no negative cultures known.   - isolation and admission culture   - isolation may be lifted at 5 negative cultures. - Last positive culture > 1 year ago and there are no negative cultures known.   - isolation and admission culture and 2nd culture after 24h   - lift isolation in case of 2 negative cultures - Last positive culture > 1 year ago and 1 negative culture known.   - isolation and admission culture   - lift isolation in case of negative admission culture - Last positive culture > 1 year ago and 2 negative cultures known.   - declare negative in case negative uptake culture. - Last positive culture > 1 year ago and ≥1 negative culture known.   - declare negative if case of 3 negative cultures. - Last positive culture > 4 years ago even no negative culture known.   - declare negative. | - Last positive culture < 1 year ago   - isolation and admission culture   - isolation is not lifted within 1 year. - Last positive culture > 1 year ago and there are no negative cultures known.   - isolation and admission culture and second culture after 24h   - lift isolation in case of 2 negative cultures - Last positive culture > 1 year ago and 1 negative culture known.   - isolation and admission culture   - lift isolation in case of negative uptake culture - Last positive culture > 1 year ago and 2 negative cultures known.   - admission culture   - declare negative in case of negative admission culture. - Last positive culture > 1 year ago and ≥1 negative culture known.   - declare negative if case of 3 negative cultures. |
| recommended PPE for HCPs | - gloves, long sleeve apron | | - gloves and disposable gown | |

*HCW: healthcare worker; NL, the Netherlands; NA, not applicable; PCR, polymerase chain reaction; PPE, personal protective equipment; SRI, Samenwerkingsverband Richtlijnen Infectiepreventie; UMCG, University Medical Center Groningen.*

^1^ for the individuals who have been to Asia and/or Africa <2 months ago without healthcare facility stays: healthcare institutions are recommended to choose to include this group in the risk inventory depending on the local situation.

^2^ contact isolation of a patient with ESBL-E may take place in a multiple room, provided that the requirement of the Board of Hospital Facilities that a space of 1.5 meters is free around the bed is met.

^3^ cultures are not reliable when using antibiotics that suppress the growth of HRMO in the 48 hours before collection.

^4^ only apply if the patient was admitted for more than 24 h or less than 24h if an invasive procedure or risk factor was present.

^5^ as long as patients live an asylum center, cultures are repeated every 2 months.

^6^ in case of specific situation: Sputum culture in intubated patients and in patients giving up sputum, Smear of wounds and skin lesions (e.g., eczema or psoriasis), Urine culture in patients with indwelling catheters or suspected urinary tract infection, umbilical smear in neonates (as long as the umbilical stump has not dried in)

**^7^** cleaning and disinfection of the room and waste are handled differently. the patient lies in a sluiced room with the doors closed, allowing better differentiation between the clean and dirty zone.

Conditions for lifting isolation in case readmission at UMCG: Only cultures taken when the patient is not on antibiotics are reliable. If the patient is on antibiotics, valid cultures can be taken at least 48 hours after stopping antibiotics. Subsequent HRMO cultures should be taken at least 24 hours apart. If a patient's readmission cultures are positive, protective measures during the relevant admission are not lifted.
